# Ability of proper application of ultrasound (US) and a fine needle aspiration biopsy (FNAB) in the management of thyroid nodules/focal lesions in patients with coexisting additional worrying clinical signs and symptoms

**DOI:** 10.1186/1756-6614-6-S2-A39

**Published:** 2013-04-05

**Authors:** Andrzej Lewiński, Zbigniew Adamczewski

**Affiliations:** 1 Department of Endocrinology and Metabolic Diseases, Medical University of Lodz, Poland; 2 Polish Mother’s Memorial Hospital – Research Institute, Lodz, Poland

## 

Application of the main principle - that therapeutic decisions should be taken only on the basis of FNAB result, while US is used to assess qualification of patients for FNAB and to decide on the selection of lesions that should be submitted to FNAB - may be associated with the risk of taking the wrong decision, based on erroneous assumptions. We must remember - especially in the era of dominance of various recommendations and guidelines, pointing to the need for thyroid US and FNAB diagnostics in every single patient with thyroid tumour/tumours, the principles of common sense in thought and deed. It is our patient and his/her future is the most important to us, and not getting the results, especially when the clinical context indicates clearly the need for urgent surgery. Repeat testing or performance of time-consuming diagnostic procedures - is a mistake. The results will not - in a majority of circumstances - alter therapeutic management but can only delay introduction of effective treatment.

We cannot forget that the patient has the right to demand surgical treatment and a doctor cannot refuse to perform surgery in all cases when the presence of thyroid lesion is confirmed - regardless of US risk patterns and FNAB results and of initial diagnosis. Despite technological development and the use of modern diagnostic equipment, basing for the time being, the diagnoses on preoperative tests, e.g. on the assignment to each TBSRTC category - does not provide 100% certainty of confirmation (positive verification) in postoperative histopathological examination.

We are aware of the fact that our proposals of optimal management, like many others developed previously in order to assess the nature of thyroid lesions, do not solve all the possible clinical problems. However, it is important to take into account the most essential features visualised in US examination which are related to an increased risk of malignancy and by considering them in combination with the results of cytological assessment. We urge that stress be laid on the importance of other potentially compounding clinical signs and symptoms and believe that our scheme of management with thyroid nodules/US focal lesions will fulfil its role in everyday medical practice and will facilitate to make the right diagnostic-therapeutic decisions.

